# Quantification of glycated IgG in CHO supernatants: A practical approach

**DOI:** 10.1002/btpr.3124

**Published:** 2021-01-21

**Authors:** Gabriele Lhota, Bernhard Sissolak, Gerald Striedner, Wolfgang Sommeregger, Karola Vorauer‐Uhl

**Affiliations:** ^1^ Institute of Bioprocess Science and Engineering, Department of Biotechnology University of Natural Resources and Life Sciences Vienna Austria; ^2^ Research and Development Bilfinger Industrietechnik Salzburg GmbH Salzburg Austria

**Keywords:** boronate affinity chromatography, CHO cell culture, glycation, post‐translational modification, recombinant monoclonal antibody

## Abstract

Post‐translational, nonenzymatic glycation of monoclonal antibodies (mAbs) in the presence of reducing sugars (in bioprocesses) is a widely known phenomenon, which affects protein heterogeneity and potentially has an impact on quality, safety, and efficacy of the end product. Quantification of individual glycation levels is compulsory for each mAb therapeutically applied in humans. We therefore propose an analytical method for monitoring glycation levels of mAb products during the bioprocess. This is a useful tool for process‐design considerations, especially concerning glucose‐feed strategies and temperature as major driving factors of protein glycation. In this study, boronate affinity chromatography (BAC) was optimized for determination of the glycation level of mAbs in supernatants. In fact, the complex matrix found in supernatants is an underlying obstacle to use BAC, but with a simple clean‐up step, we found that the elution profile could be significantly improved so that qualitative and quantitative determination could be reached. Complementary analytical methods confirmed the performance quality, including the correctness and specificity of the results. For quantitative determination of mAb glycation in supernatants, we established a calibration procedure for the retained mAb peak, identified as glycated antibody monomers. For this approach, an available fully characterized mAb standard, Humira®, was successfully applied, and continuous monitoring of mAbs across three repetitive fed‐batch processes was finally performed. With this practical, novel approach, an insight was obtained into glycation levels during bioprocessing, in conjunction with glucose levels and product titer over time, facilitating efficient process development and batch‐consistency monitoring.

## 1 INTRODUCTION

Post‐translational modifications of therapeutically relevant proteins play a decisive role due to their distinct properties and functions and should therefore be monitored carefully. Besides glycosylation (the most prominent intracellularly processed modification), extracellular events also lead to significant post‐translational modifications of the protein.^1^ In this respect, nonenzymatic chemical modification of proteins (the glycation of N‐terminus and each potential lysine sidechain, respectively) in the presence of reducing sugars (aldose or ketose) leads, in the final phase, to the formation of irreversible advanced glycation products.^2^, ^3^ In the food and pharmaceutical industries, glycation has become of great significance in relation to several therapeutic candidates, especially recombinant monoclonal antibodies (mAbs).^4^ Although the glycation cascade has been thoroughly investigated, few studies have explored bioprocess‐relevant protein glycation. In fact, numerous studies have focused on elucidation of the glycation mechanism, the effect of reducing sugars in purified protein formulations and development of analytical methods to study this phenomenon.^5^, ^6^, ^7^ Less attention has been paid to the formation of glycated mAb during bioprocess development.^8^ However, the few bioprocess‐related studies that have been carried out describe the significant impact of environmental conditions, salts and media components.^9^, ^10^, ^11^ These studies were primarily performed by forced glycation experiments in vitro, while the mAb was purified, mostly by Protein A chromatography prior to analysis. These studies are of importance for the investigation of glycation levels and individual glycation sides, which are essential for the final formulation and storage of a defined product.

Besides chromatographic methods involving mass‐spectrometric detection (MS), measuring the relative abundances of mono and higher order glycated isoforms, boronate affinity chromatography (BAC), including DAD detection, is widely used to determine overall glycation levels of mAbs.^12^ BAC is based on the highly specific and reversible interaction between the tetrahedral anion that forms from boronic acid at an alkaline pH and *cis*‐1,2‐diol structures that are found in sugar molecules. Glycated protein molecules contain these additional sugar structures in the open‐chain form, and they interact with the boronate ligand. Elution is obtained by lowering the pH to disrupt the interaction or by competition induced via an additional source of hydroxyl groups, such as sorbitol buffer. ^10^, ^13^ Chromatographic methods with subsequent MS detection exhibit a notable drawback, that is, that sample preparation is mandatory and laborious regarding the complex matrix that exists in culture supernatants. Additionally, there is an immanent risk of glycation linkages being affected during this step, leading to underestimation of glycation levels. ^12^, ^14^

However, from a quality‐by‐control (QbC) perspective, an in‐depth understanding of glycation formation during a bioprocess is advisable, since it would enable to control glycation beyond a critical level.^8^, ^15^ QbC enables definition of the design space within which a process is controlled. In terms of this task, a reliable set of quantitative data must inevitably be analyzed using a robust analytical method. To overcome the analytical burden, methods involving a minimal preparatory workload should be applied.

To the best of our knowledge, BAC analyses for the determination of glycation levels of proteins are solely performed with highly purified mAbs.^9^ BAC analysis involving alternative timesaving techniques directly performed in supernatants could not be found in the available literature. The particular challenge of an alternative procedure is obvious due to the complexity of supernatant composition. Host‐cell proteins, as well as the target protein itself and its potential aggregation, may alter the quality of the results.^10^ Furthermore, low‐molecular‐weight molecules may also interfere with the chromatographic result. To overcome these limitations, optimized chromatographic conditions and appropriate samples are required to avoid unspecific binding.

In this study, we focused on a practical approach for determining glycation levels of mAbs in a complex supernatant matrix. The antitumor necrosis factor (anti‐TNF‐α) IgG1 (recombinantly produced in Chinese hamster ovary cells in a fed‐batch process) was used as a model protein for our investigations to prove the feasibility of an alternative monitoring concept for the determination of glycation levels in bioprocesses. As convenient standard, a highly purified and fully characterized mAb, Humira®, was purchased. Matrix effects, chromatographic conditions, sample preparation opportunities and necessities were systematically assessed, and complementary methods were used to evaluate the correctness and applicability of our procedure. The following were found to constitute a convenient and accurate bioprocess monitoring tool for accurate determination of glycation levels: simple sample preparation to separate low‐molecular‐weight molecules; thoroughly adjusted chromatographic conditions; and the selected calibration design.

## 2 MATERIALS AND METHODS

### 2.1 Materials and reagents

All chromatographic analyses (except Protein A chromatography) were conducted on a Series 1200 Agilent HPLC system equipped with a vacuum degasser, a binary pump, autosampler and DAD detector. Chromatograms were evaluated using Agilent Chemstation software (revision B.04.01).

Humira® an anti‐TNF‐α IgG1 was used as mAb standard (Humira^TM^, Adalimumab AbbVie). N‐(2‐Hydroxyethyl) piperazine‐N′‐(3‐propanesulfonic acid) (EPPS) and sorbitol were purchased from Sigma‐Aldrich, and phosphate‐buffered saline (PBS), Tris base, NaCl, Na_2_HPO_4_.2H_2_O, NaH_2_PO_4_.2H_2_O, Tween, glycine, Tris HCl, guanidine HCl, and sulfuric acid were obtained from Merck‐Millipore. All chemicals were of pro analysi grade. Sample filtration was performed with a 0.22 μm syringe filter (La‐Pha‐Pack), and sample clean‐up was carried out with illustra Nap‐5 columns (GE Healthcare).

### 2.2 Boronate affinity chromatography

Samples (100 μl injection volume) were analyzed on a TSKgel Boronate‐5PW column (7.5 mm × 75 mm) (Tosoh Bioscience, Montgomeryville, PA). Column temperature was set to 40°C at a flow rate of 1 ml/min. The mobile phases used were as follows: A: 50 mM EPPS, 10 mM Tris, 200 mM NaCl at pH 8.3; and B: 500 mM sorbitol in mobile Phase A. Elution was conducted isocratically with mobile Phase A for 20 min, followed by a gradient to 100% B in 5 min; 100% B was held for 5 min, with subsequent re‐equilibration with mobile Phase A for 10 min. Detection was performed at 280 nm. Samples were 0.2 μm filtrated prior to injection or applied on a Nap‐5 column as a sample clean‐up step for the removal of salts and small molecules up to 5 kD.

### 2.3 Calibration standard

For calibration of the glycated protein moieties, a stock solution of the highly purified mAb standard (1 mg/ml) was diluted in PBS to different concentrations of 800, 600, 400, 200, and 100 μg/ml. The applied standard material, Humira®, one of the best characterized mAb was placed at our disposal for calibration and BAC method optimization.

### 2.4 Size exclusion chromatography

Size exclusion chromatography (SEC) analyses were performed on a TSK G3000SW column (7.8 mm × 300 mm) (Tosoh Bioscience) at 25°C and a flow rate of 0.8 ml/min. After sample injection (20 μl), separation was obtained isocratically with a 100 mM sodium phosphate buffer (pH 6.7). The detection wavelength was 214 nm.

### 2.5 Protein A chromatography

MAb purification from clarified supernatants was performed on an Äkta Pure system (GE Healthcare). A POROS A 20 (2.1 mm × 30 mm) column (Thermo Scientific) was equilibrated with phosphate buffered saline (PBS) pH 7.4 for 25 column volumes (CVs); 2 ml of supernatant was loaded with subsequent washing of the column with PBS for 20 CVs. Elution of the mAb was conducted with 100 mM glycine buffer, pH 3.0 in a 10 CVs step gradient, and collected fractions were neutralized with 1 M Tris HCl, pH 8.0. Column cleaning was performed with a solution of 6 M guanidine HCl and 50 mM Tris, pH 8.0 for two CV and re‐equilibrated with PBS. Detection was performed at 280 nm. Due to tailing of the eluting mAb peak, only the main portion of the peak was collected, resulting in 5% material loss.

### 2.6 Bio‐layer interferometry)

Product titer in culture supernatants was determined by bio‐layer interferometry (BLI) with the Octet QK system (Pall Forte Bio), as previously described by Sissolak et al.^16^ Briefly, samples were diluted in phosphate buffered saline with 0.1 vol% Tween 20 (PBS‐T) and transferred to each well of a black, nonsterile 96‐well plate (Thermo Fischer Scientific). Protein A biosensor tips were equilibrated in PBS‐T, and plates were shaken at 1000 rpm for 5 s before each measurement. The binding rates of mAb to Protein A were determined over a time interval of 300 , and mAb quantity was calculated via a calibration curve in the concentration range between 10 and 50 μg/ml. Calculation was performed using Octet software (version 6.4, ForteBio).

### 2.7 Ion exclusion chromatography

For glucose quantification in supernatants, a HPX‐87H 300 × 7.8 mm column (Bio Rad) tempered at 25°C was used. The mobile phase consisted of 5 mM sulfuric acid, and the flow rate was set to 0.45 ml/min. Detection was performed with a refractive index detector (Agilent) at 35°C. Samples were filtered and diluted within the calibration range of d (+) glucose (100–2000 mg/l). The injection volume was 20 μl.

### 2.8 Bioprocess design

As a model protein, anti‐TNF‐α IgG1 (Adalimumab) was used, produced by a recombinant monoclonal CHO cell line (Antibody Lab GmbH, Austria). Generation of the cell line was conducted by applying the Rosa26 bacterial artificial chromosome (BAC) expression strategy to a serum‐free adapted host cell line derived from CHO‐K1 (ATCC CCL‐61).^17^ The procedure for cell‐line propagation and the parameters for the fed‐batch process in 15 L scale have previously been described by Wallner et al.^18^ The experimental setup, including temperature (37°C, switched to 34°C on Day 3) and feed strategy (constant), was kept identical for every three consecutive experiments shown in this article. As batch medium Dynamis AGT (Thermo Fisher) was used. Additional 20 g/L glucose (G7021; Sigma‐Aldrich, Germany) and 0.1% antifoam (A8011; Sigma‐Aldrich) were added to the feed media (CHO CD EfficientFeed A, A1442001; Thermo Fisher Scientific). Levels of dissolved oxygen (DO) were kept above 30%, and the pH was maintained constant at pH 7 via CO_2_ sparging.

The mock control experiment was performed with the nonproducing host cell line and was carried out in a shake flask, as described by Sissolak et al.^16^

## 3 RESULTS AND DISCUSSION

### 3.1 BAC for in‐process monitoring

Further development and optimization of a BAC‐HPLC method which is convenient for in‐process monitoring of mAb glycation levels requires several steps. The initial step is optimization of the BAC method, adapted to the physicochemical properties of the mAb to be analyzed.^11^ Therefore, a highly purified and fully characterized mAb standard is required for qualitative and quantitative improvements. As already published, optimization of the mobile phase is conducted by the addition of Tris as a shielding reagent, NaCl to suppress electrostatic interaction and varying the pH value. ^10^, ^11^ Variable concentrations in the loading and elution buffer, to suppress non‐specific interactions between mAb and the boronic acid ligand (which would lead to overestimation of glycated protein variants), need to be tested.

**FIGURE 1:**
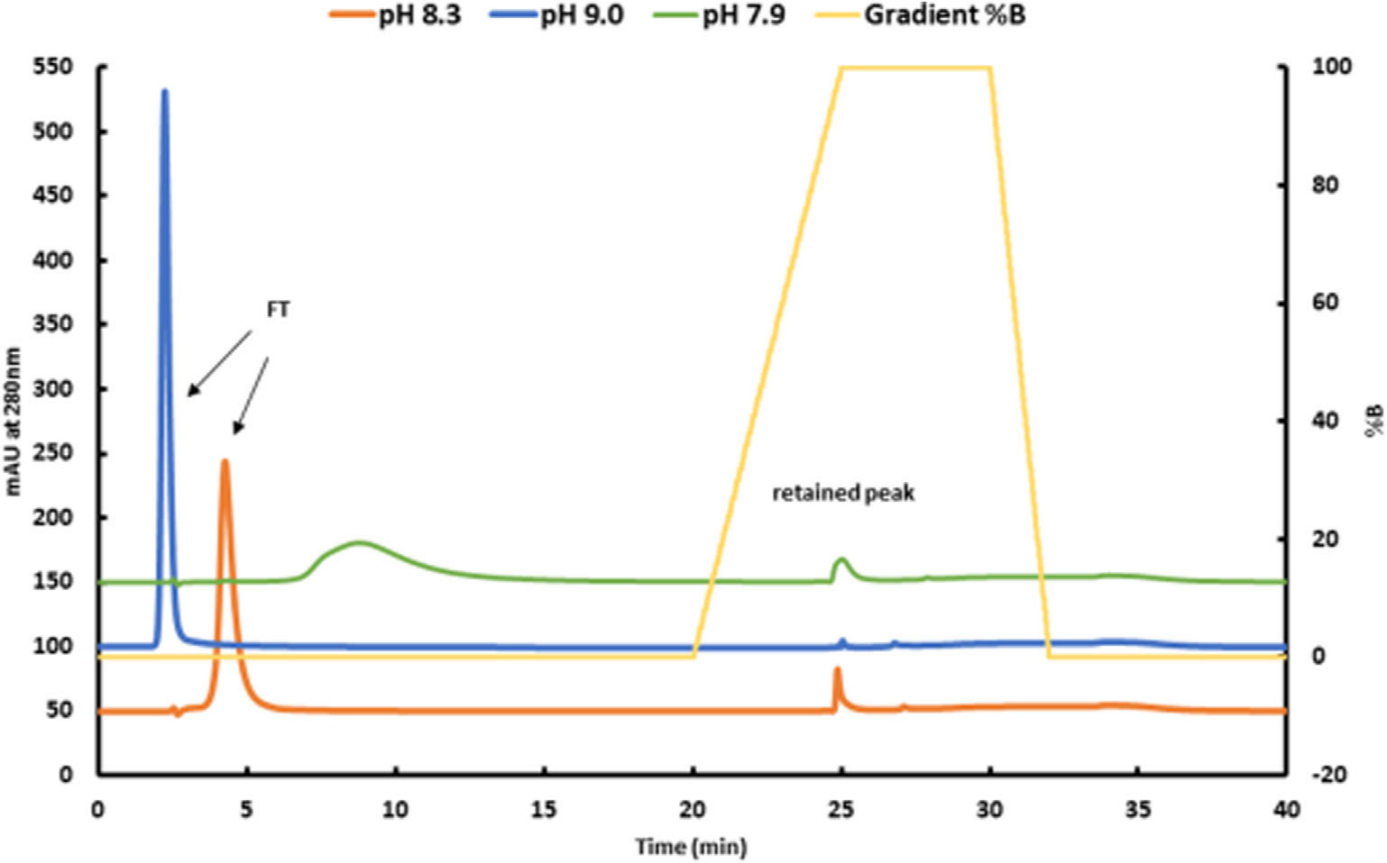
Boronate affinity chromatography of the mAb standard (Adalimumab) at pH 8.3, 9.0, and 7.9 of the binding (a) and elution buffer (b). Buffer A was 50 mM EPPS, 10 mM Tris, 200 mM NaCl, and buffer B was 500 mM sorbitol in buffer A. FT marks the flow through of unbound protein variants while the retained peak represents the glycated fraction

For the mAb standard, Humira®, 10 mM of Tris and 200 mM of NaCl were found to be appropriate to avoid unspecific interactions between mAb and boronate affinity resin. PH variations from 7.9 to 9.0 in the buffer system showed the significantly different flow‐through (FT) and binding behavior (retained peak) of this standard (Figure 1). PH 9.0 completely inhibited interaction between the standard‐protein and column resin, indicating that only the FT peak was detected, with no retained peak. On the other hand, pH 7.9 provoked complete unspecific binding of the mAb onto the column surface. In between these extreme ranges, pH 8.3 was identified as being the optimal pH value for this particular mAb, achieving the same glycation level as specified in the standard data sheet and is in good agreement with results of various Humira® batches of United States and European origin. Interestingly, for the BAC in the literature, clear strategies can be found for optimization of Tris and NaCl concentrations in the solvents.^10^ However, less detailed information is available regarding the pH optimum for the solvents. So far, the correlation between the isoelectric point (pI) of the mAb and the pH optimum of the buffers has not been studied in detail. Based on our data, we suppose that both the buffer composition and the pH have a significant impact on binding behavior of the analyzed mAb. Selective binding of *cis*‐diol groups operates under basic conditions, meaning that boronate carries a negative net charge. At pH 9.0, the analyzed mAb is also negatively charged overall (pI 8.4), and it seemed that due to electrostatic repulsion, no interaction could take place. Additionally, complete mAb binding at pH 7.9 indicated that the net charge of the analyte, which is highly positive, had a distinct contribution to binding behavior onto the column. In contrast, the only slightly positive net charge of the protein at pH 8.3 enabled binding. Although Zhang et al assume that the individual pI value of the mAb does not play a critical role in binding to the boronate column, our results clearly indicate that the pH value needs to be thoroughly adjusted in combination with the salt concentrations.^11^, ^19^

For the standard experiments, glycation levels were determined, as conventionally proposed, by calculating the percentage of the integrated peak area of the retained peak versus total peak area. The resulting glycation level of 6.2% of the standard protein was within the expected glycation range of the Humira® confirming that pH 8.3 was appropriate for analysis of our product.^20^ The optimized method was shown to be robust and reproducible, which is reflected in the 93.8% mean value with a 3.0% relative standard deviation for the FT peak area units and 6.2% mean value, and a 5.3% relative standard deviation for the retained peak area units (triplicate measurements on three different days).

### 3.2 BAC in complex matrices

**FIGURE 2:**
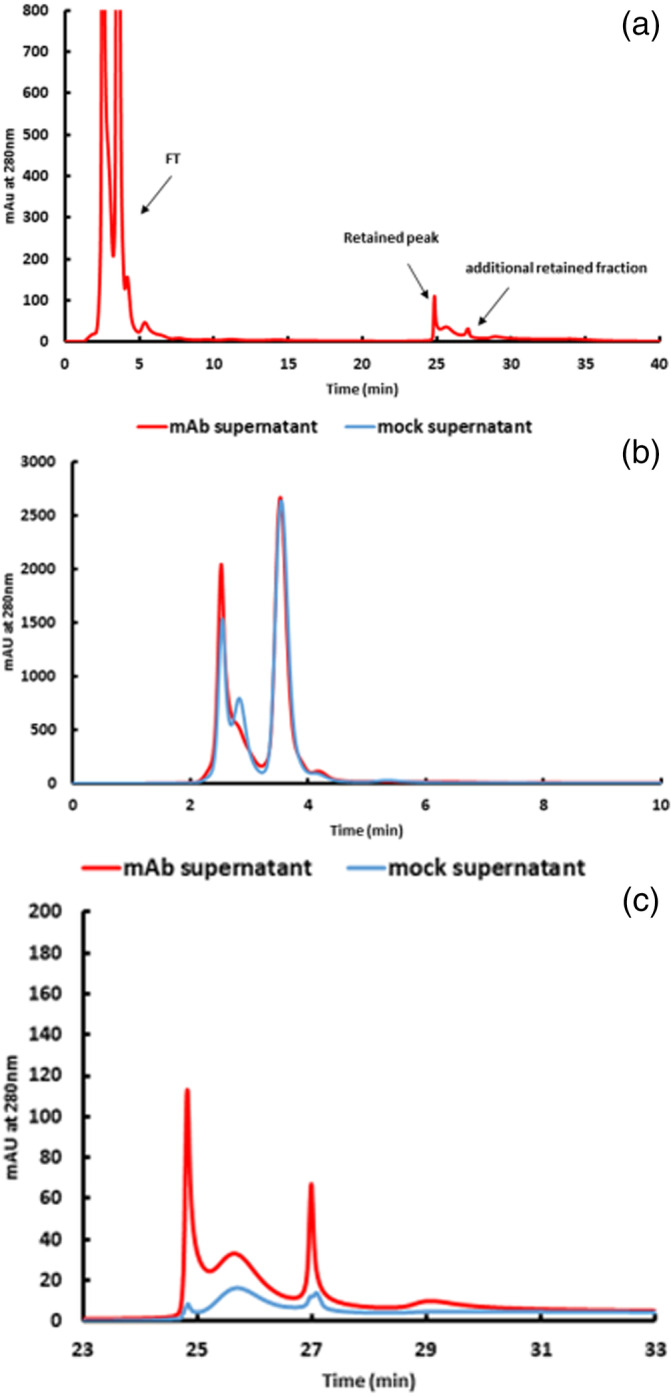
(a) Peak profile of boronate affinity chromatography of mAb containing, undiluted supernatant (0.2 μm filtrated) yielded from a CHO fed‐batch culture without any further sample preparation. Chromatographic conditions were the same as in Figure 1 at pH 8.3. (b) Detailed comparison of flow through elution profiles of 0.2 μm filtrated supernatants of a mAb producing CHO fed‐batch and a mock fed batch, without mAb product. (c) Detailed comparison of retained area elution profiles of supernatants of a mAb producing CHO fed‐batch and a mock fed batch, without mAb product

BAC is widely used for determining the glycation level of purified mAbs. However, monitoring of the glycation status of mAbs during the fermentation process is of high relevance as this important quality attribute has a significant impact on the final product quality and yield.^4^ It is well known and widely discussed in the literature that glucose concentration, temperature, product titer and process duration significantly affect the glycation level of the product. ^21^ This circumstance is crucial in process development for fed‐batch fermentation but even more important for continuous processing, monitoring of existing processes and also for downstream development, in order to consistently purify high‐quality products and avoid preventable product loss.^9^ Thus, in conclusion, early insight into this post‐translational modification process is a facilitating tool for efficient fermentation process development and optimization. From an analytical perspective, our studies were focused on influences falsifying the analytical result by interfering compounds in the samples. Therefore, a harvested supernatant was only 0.2 μm filtrated and analyzed; a representative chromatogram is shown in Figure 2a. As expected, conventional calculation of glycation levels of the mAb product in supernatants was inappropriate due to the presence of, for example, host‐cell proteins, DNA, and aggregates. The lack of a defined FT peak and the unacceptable peak shape for integration of the retained peak (due to the increasing baseline), as well as the unidentified additional retained fractions, required a different approach for monitoring of glycation levels in complex matrices. In parallel, we analyzed the supernatant of a mock fed‐batch which did not contain any mAb product. By comparing the elution profile of the supernatant with the mock supernatant, it became obvious that the FT profile was almost the same, meaning that the unglycated mAb peak was overlapped by signals caused by matrix proteins (Figure 2b). In contrast, at the expected retention time of the glycated fraction, only an insignificant signal and, subsequently, a slight baseline drift were seen (Figure 2c). This suggests that no significant unspecific interaction between supernatant matrix molecules and the affinity column surface was evident. However, injection of the crude supernatant (without any sample preparation) may potentially falsify the results because no precise integration of the retained peak is feasible. Therefore, a simple sample clean‐up step was applied. This clean‐up step was able to eliminate small compounds that were not relevant to the product, up to 5 kD, while the mAb product in the supernatant remained untouched.

**FIGURE 3:**
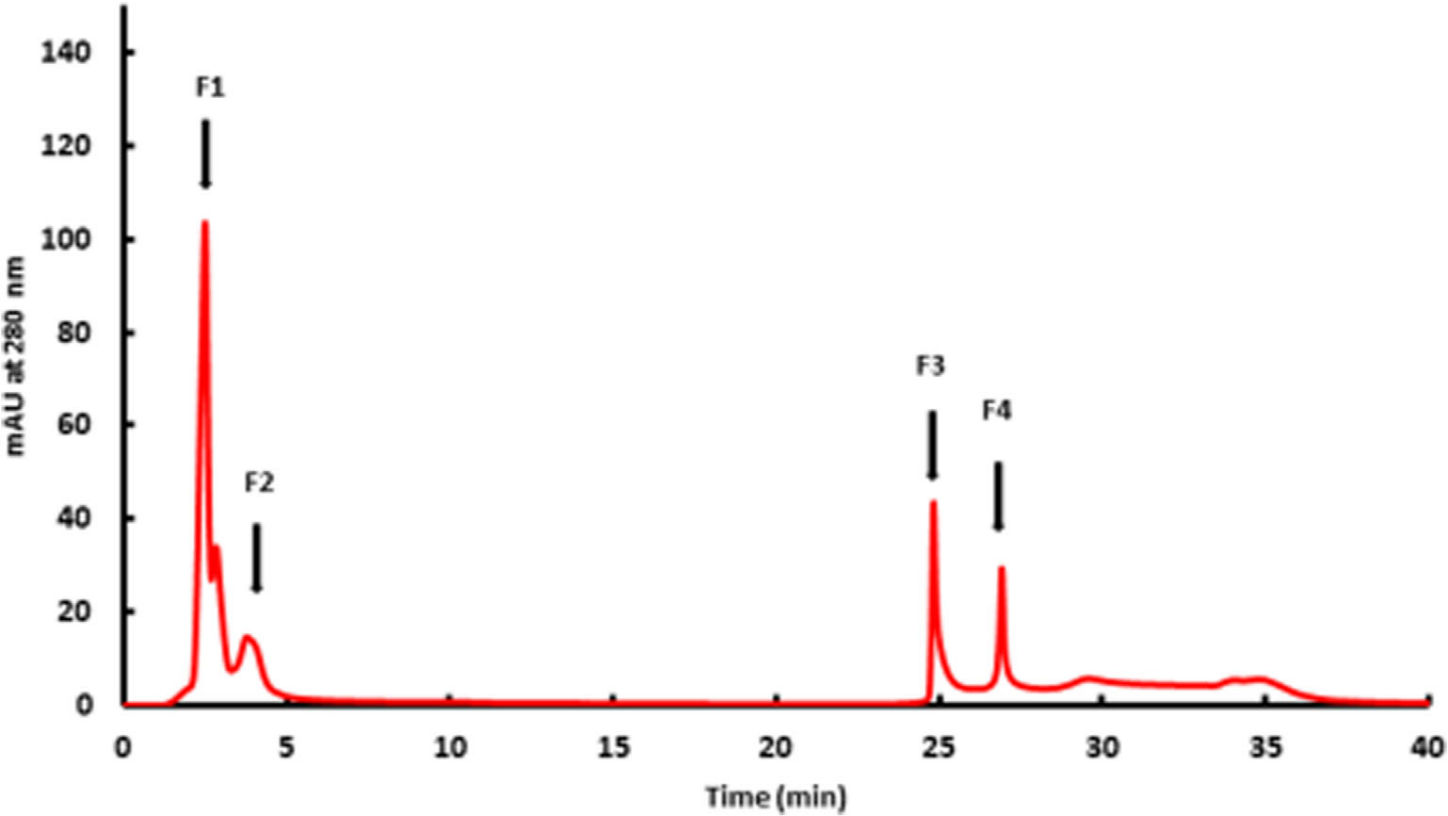
Boronate affinity chromatogram of a Nap‐5 eluate of a CHO fed‐batch mAb supernatant. Marked fractions (F1‐F4) were collected for size exclusion chromatography for further analysis

Removal of low‐molecular‐weight compounds from the supernatant was performed on Nap‐5 columns. In accordance with the manufacturer's instructions, the elution was performed with PBS buffer. Analysis of the Nap‐5 eluates by BAC‐HPLC showed a significant improvement in the elution profile (Figure 3). A distinct rearrangement in the FT profile of the supernatants was obvious, followed by two clearly separated retained peaks. This evidences that a high percentage of the unassignable signals originated from low‐molecular‐weight molecules. Nevertheless, the ambiguous FT and the additionally bound fraction after the glycated peak were not suitable for conventional assessment and/or calculation of the glycation level of the mAb. The lack of small molecules could be confirmed by size exclusion chromatography. Analysis of Nap‐5 eluates and simply filtrated supernatants showed that IgG content remained unaffected, while small molecules were eliminated via Nap‐5 (supplement S1a and S1b). A similar clean‐up procedure was performed with the mAb standard, demonstrating that neither product‐relevant alteration nor product loss during this step occurred (supplement S2a and S2b). Consequently, systematic elucidation of the FT fraction and the two retained fractions was mandatory. For further investigation, the individual BAC peaks (F1–F4), as depicted in Figure 3, were collected and analyzed with SEC as a complementary technique.

### 3.3 Peak identification by SEC

**FIGURE 4:**
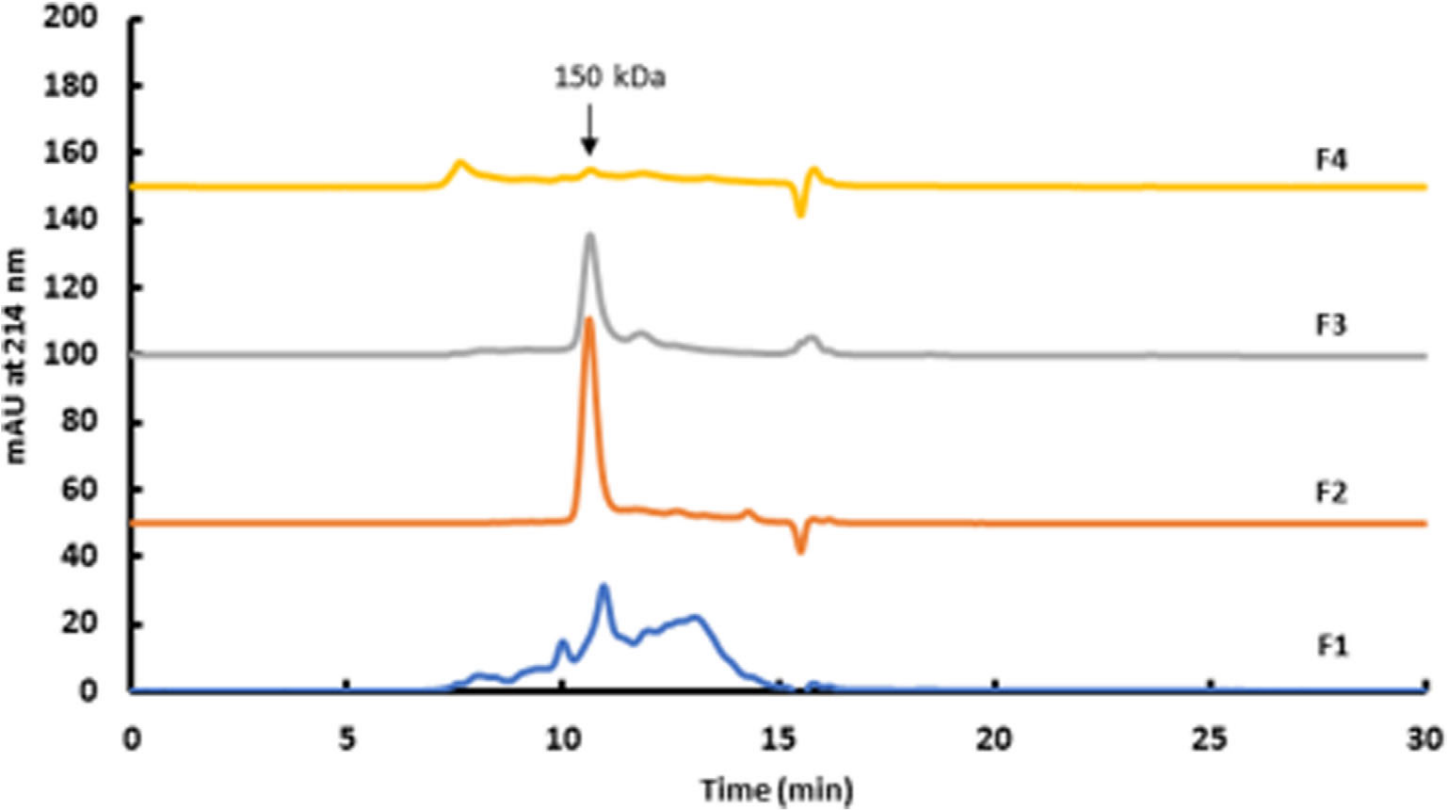
Size exclusion chromatography with a 100 mM sodium phosphate buffer, pH 6.7 of collected boronate affinity chromatography (BAC) fractions F1–F4 as indicated in Figure 3. F1: various populations of molecules in a broad MW‐range. F2,F3: IgG monomers. F4: high‐order aggregates The individual molecular weights were assigned using a molecular weight standard

Although it would be preferable if only antibody monomers were expressed, it is well known that minor amounts of aggregates are almost always present in culture supernatants.^22^ In the case of the boronate affinity method, this circumstance is relevant because, as postulated in the literature, mAb aggregates could be co‐retained on the column and lead to overestimation of the glycation level. Therefore, it is suggested that the aggregates be removed.^10^ For further investigation, the collected fractions from BAC were analyzed with size exclusion chromatography to prove potential interferences. As shown in Figure 4, the BAC flow‐through (F1 and F2; marked in Figure 3) clearly indicates the presence of higher order and dimeric aggregates in F1 as well as intact IgG (monomer), followed by IgG monomers without aggregates in F2. The two retained peaks that were collected were identified as intact IgG fraction F3, while F4 contained only high‐order aggregates. These findings evidence that under optimized chromatographic conditions, no interference of glycated IgG monomer and aggregates occurred. Additionally, specific binding of the native glycated IgG at the expected, determined retention time was seen. Furthermore, under the optimized chromatographic conditions, IgG aggregates were found to associate with the resin but could be eluted differently. The binding behavior of mAb aggregates to boronate affinity columns has been discussed extensively in the literature.^11^ However, it has not been evidenced that they might be separated from the glycated fraction and that, by this co‐elution of aggregates, overestimation of the glycation level is caused (if there is at least one glycation unit on them).^11^ This fact implies that aggregates are retained contemporaneously with glycated mAb versions. Similarly, Quan et al postulate increased interaction of aggregates with the boronate chromatographic resin and suggest thoroughly controlling the quality of the samples.^10^ As shown in Figure 3, under optimized chromatographic performance, aggregates of this mAb (F4) seem to exhibit prolonged retention behavior, compared to glycated monomeric mAb, which is an indication of at least one glycation unit on them. The contemporaneous presence of aggregates in the FT, which are consequently unglycated, supports this assumption. With our approach to monitoring the glycation levels of a mAb product during the bioprocess, the different retention time of aggregates is significant because the binding of relevant monomeric glycated structures is not affected. In conclusion, the established method (based on a known separation mechanism) offers several additional benefits, compared to existing strategies, notably simple, robust and lossless workflow for culture supernatants and adequate resolution, with clear evidence that the amount of glycated native IgG can be reliably quantified.

### 3.4 Method verification with Protein A purified mAb

**FIGURE 5:**
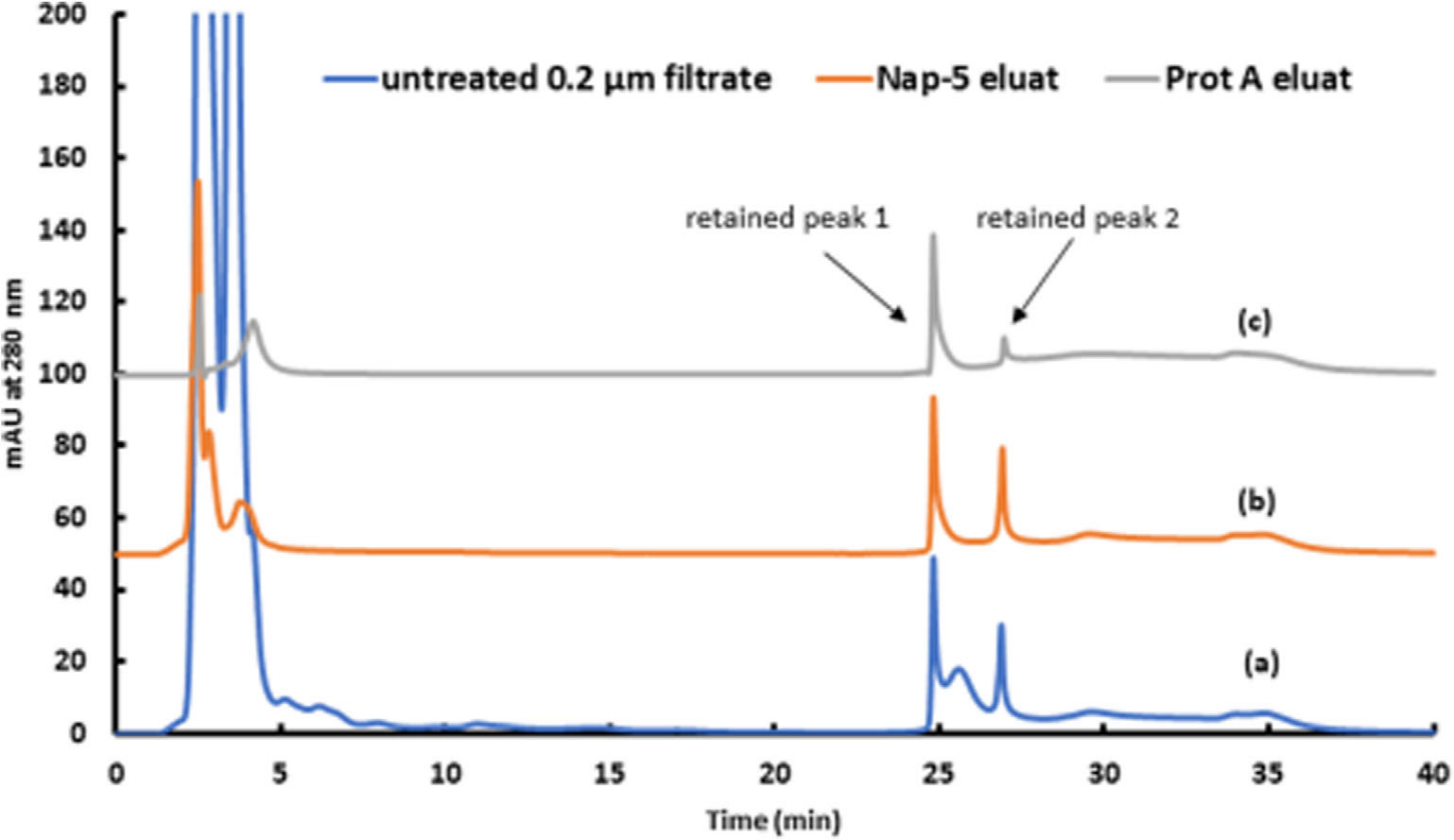
Comparison of the 2.0 μm filtrated supernatant (a), the Nap‐5 eluate, used as clean‐up procedure (b) and the mAb, purified via Protein A (c)

In a control experiment, a standardized capture step for clarified supernatant was performed using Protein A chromatography, followed by BAC analysis. The fact that this technique binds the native mAb as well as aggregated mAb was reflected in subsequent BAC analysis.^23^ Figure 5 shows a comparison of untreated supernatant (a), a Nap‐5 eluate (b), and the Protein A eluate (c). It is clear that the flow‐through profile varies between the total matrix of untreated supernatant (a), the matrix without low‐molecular‐weight compounds (b) and IgG captured by Protein A. However, more importantly, the specific retained peak for the glycated fraction of interest was unaffected, irrespective of which sample preparation method was applied. Nevertheless, the unspecific retained fraction that followed was significantly reduced in the Protein A sample because during Protein A capture, the quantity of aggregates was significantly reduced. Thus, by collecting only the main portion of the peak, aggregates were separated. This control experiment further confirmed that the mAb aggregates could be bonded but did not interfere with the aspired glycation fraction.

### 3.5 Forced antibody aggregation

**FIGURE 6:**
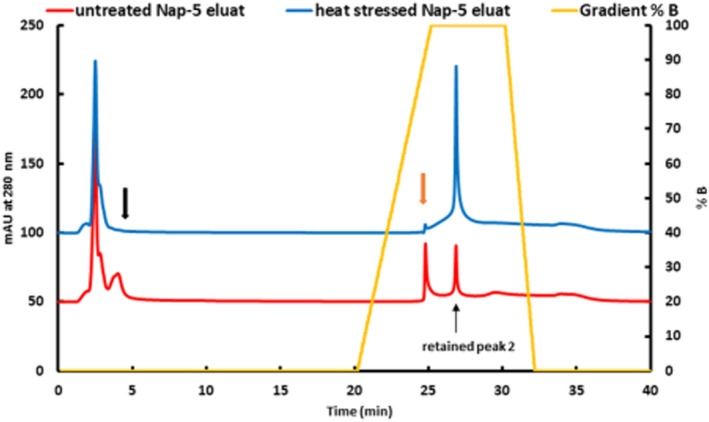
Boronate affinity chromatography (BAC) analysis of an untreated, Nap‐5 eluate of a supernatant and the corresponding heat stressed Nap‐5 eluate (4 hr, 70°C). Heat stress‐induced IgG aggregation causes loss of unglycated IgG monomers in the flow through (black arrow) and glycated IgG monomers, (orange arrow). Retained peak 2 increases significantly, indicating the separation of aggregates

Although SEC and Protein A control experiments strongly indicated the reliability of our results, we wanted to substantiate these indications. Therefore, forced antibody aggregation was performed to get a better insight into binding behavior using BAC. Through this experiment, we intended to study the BAC elution profile when almost all the applied mAb was aggregated. A forced IgG aggregation was created by heat stress, with the Nap‐5 eluate being exposed to 70°C for at least 4 hr, cooled to room temperature and immediately analyzed by SEC and BAC.^24^ SEC results clearly indicated the formation of large oligomers associated with a complete loss of the native protein (data not shown). Comparison of the elution profile of an untreated and heat‐stressed sample by BAC analysis is shown in Figure 6. Heat stress led to a loss of unglycated IgG in the FT (marked with a black arrow) and specifically retained glycated IgG (marked with an orange arrow), whereas the unspecific retained fraction increased significantly. This fact supports the assumption that aggregates bind to the boronate resin but can clearly be separated from the glycated fraction of interest. The plotted gradient demonstrates that glycated IgG eluted during the increase to 100%B, whereas aggregates eluted with 100%B.

As a result of our investigation of BAC performance, in terms of the applied mAb, we postulate that glycated, homodimeric mAb elutes at the expected retention time, whereas unspecific binding of aggregates occurs at a distinct retention time. Our findings indicate that BAC analysis under optimized conditions is appropriate for measuring the amount of glycated mAb, but it is not able to distinguish between the amount of glycated and unglycated aggregates due to overestimation of the measured signal.

### 3.6 Quantification of glycated mAb

**FIGURE 7:**
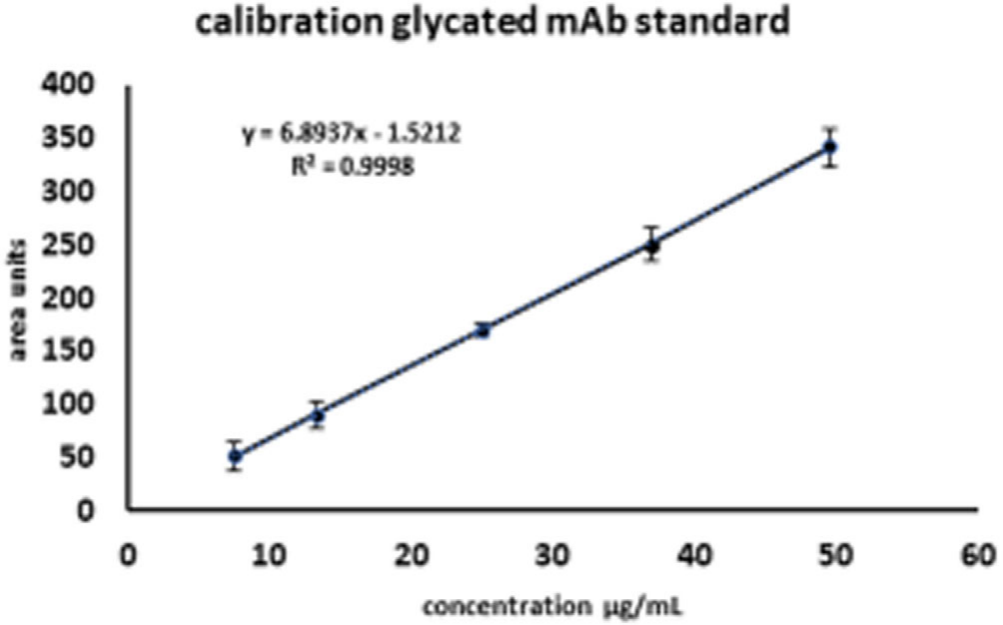
Calibration function of glycated mAb. Each concentration was injected in triplicate. Resulting standard deviation is shown in error bars. Calculated LOD is 3.95 μg/ml and LOQ is 13.26 μg/ml

After method optimization and identification studies, calibration of the method was performed. In terms of the relative glycation of mAb, with conventional calculation, a defined and clearly integrable flow‐through and retained peak are required. This prerequisite is not ensured in complex matrices. As illustrated, FT of supernatant samples is heterogenous, and the peak area associated with IgG is not exactly definable. However, based on the optimization experiments, it can be assumed that the specifically retained peak on the boronate affinity resin solely contained the glycated IgG species that we were interested in. Additionally, it could be shown that a clean‐up step for the removal of low‐molecular‐weight compounds significantly improved the peak form of the glycated IgG. This, together with the availability of the fully characterized standard, facilitated calibration of the glycated fraction. Therefore, dilution of a series of Humira® with precisely defined concentrations (800, 600, 400, 200, and 100 μg/ml) were injected in triplicate, and the concentrations of glycated IgG were calculated as follows: First, the percentage of the integrated peak area of the retained peak versus total peak area was calculated. The second step entailed calculating the concentration of the retained peak in μg/ml, as follows:

(1) $$C_{\text{glycated}}=\frac{C_{\text{injection}}.{\%}_{\text{glycated}}}{100}$$

where *C*_glycated_ and *C*_injection_ represent the glycated and the injected concentration, and %_glycated_ describes the ratio of the retained peak to the unretained peak. A prerequisite for this approach is that the IgG glycation level is constant and not affected by the injected standard concentration (Table 1). The resulting calibration function is shown in Figure 7. By using this calibration strategy, the absolute and relative amount of glycated IgG in different supernatant samples can be calculated, and the presupposed mAb titer in the different samples is known.

**TABLE 1:** Summary of mAb standard analysis for calibration of retained peak

mAb standard	Area units^a^			Area %	Glycated mAb
μg/ml	FT	retained	Area sum	retained	μg/ml
800	5183.4	341.3	5524.7	6.2	49.5
600	3838.4	250.7	4089.1	6.1	36.9
400	2549.1	169.8	2718.8	6.2	25
200	1265.4	90	1355.5	6.6	13.2
100	646.8	51.2	698	7.3	7.5

^a^ Average of triplicate measurements.

### 3.7 Glycated mAbs in bioprocess samples

The focus of this study was to monitor product glycation in the bioprocess of a well‐characterized mAb as a model protein. Therefore, the supernatants of three fed‐batch cultivation runs were analyzed to evaluate the suitability of our analytical method under real conditions.

Due to the fact that the extent and kinetics of mAb glycation are strongly affected by the fermentation process conditions but also by the amino acid composition of the mAb and by the concentration of the expressed monoclonal antibody qualitative and quantitative analysis is relevant.^4^, ^10^, ^14^

The cultivation was performed under defined, controlled conditions. Temperature shift and glucose feeding were performed as described in the bioprocess design and were identical for each run. The stop criterion for each process was when a 70% viability threshold was reached. A sample was taken each day and analyzed.

The product titer for each supernatant sample was determined by BLI. The values for the particular batches ranged from 3 to 611 μg/ml, 2 to 748 μg/mL, and 3 to 907 μg/ml, respectively.

Nap‐5 clean‐up eluates were analyzed by BAC in triplicate, and the concentrations of retained glycated mAb were calculated, according the established calibration function described above. Standard deviation for each triplicate measurement ranged from 0.1 to 4.9. The percentage glycation of each sample was calculated and ranged from 10.2% up to 30.6%.

**FIGURE 8:**
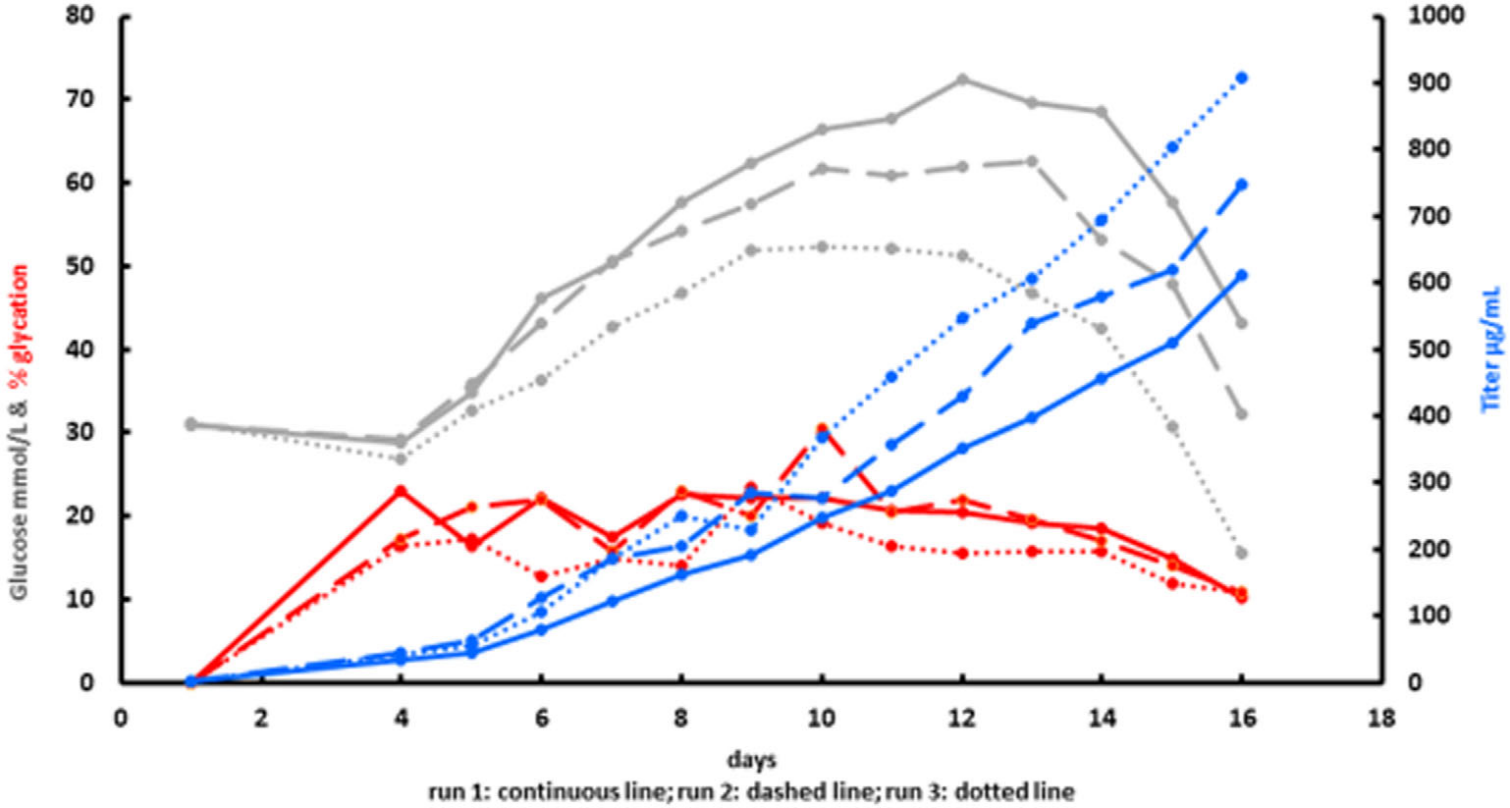
Data of three fed‐batch experiments (Run 1 continuous line; Run 2 dashed line; and Run 3 dotted line) with the volumetric mAb concentration in μg/ml (blue line), glucose concentration in mmol/L (gray line), and corresponding glycation in % (red line) are summarized. Standard deviations were between 0.06 and 4.28 (Run 1), 0.17 and 3.86 (Run 2), and 0.07 and 4.90 (Run 3) and are not shown explicitly in the graphic for reasons of clarity

Additionally, the corresponding glucose concentrations were quantified with ion exclusion chromatography. The pulse feeding started at Day 3 and lasted until Day 13, which resulted in a linear feed of 33 vol% (v/v) with respect to the end volume. Although the same feeding strategy was applied and a constant cell number was seeded, the glucose concentration in the supernatants varied in a certain extend. (see Figure 8).

Figure 8 summarizes all these data. The three cultivation runs are numbered with (1–3) which show the formation of mAb glycation levels (red line) the corresponding measured glucose concentration (gray line) and the product titer (blue line). The glycation level within the first 4 days has been qualitatively determined due to the temperature shift at Day 3. Between Days 4 and 6 semi‐quantitative glycation levels are depicted due to the low mAb concentrations. Since Say 7 quantitative calculation was possible, as seen in Figure 8.

It became obvious that different glucose concentrations are available for glycation of differently expressed antibodies. As seen in Figure 8, in Run 3 (dotted line) less glucose for a local antibody concentration was observed, and in contrast in Run 1 (continuous line), the opposite constellation was evident. However, interestingly for each batch, the profile of % glycation of the three end products was quite similar, although different antibody‐glucose ratios were determined. Furthermore, it is seen that also relatively low glucose concentrations and short cultivation times are effective in mAb glycation. Thus, our results evidenced that process monitoring is recommendable for deeper process understanding and optimization of certain bioprocesses. In this respect, simple, robust and fast sample preparation supports intensified monitoring activities.

## 4 CONCLUSION

BAC is a widely accepted procedure, which can successfully determine the glycation levels of purified mAbs. This approach cannot be employed for mAbs in complex matrices such as cell‐culture supernatants due to the lack of a defined and/or heterogenous flow‐through. Here, we propose an alternative at‐line monitoring approach, which can facilitate identification of the glycation levels of mAb products and monitoring of bioprocesses. With the robust clean‐up procedure via a Nap‐5 column, we have demonstrated that BAC is an appropriate method for direct quantification of the glycation content of culture supernatants, without further purification and consequent loss of material. As soon as highly purified and characterized standard, similar to the analyzed product, is available, separation and quantification can be established. Thus, in conclusion, the proposed procedure is useful for the intended monitoring purpose, while detailed side‐specific glycation studies should be performed differently and is outside the introduced scope.

## AUTHOR CONTRIBUTIONS

**Gabriele Lhota:** Investigation; writing‐original draft. **Bernhard Sissolak:** Investigation; writing‐review and editing. **Gerald Striedner:** Writing‐review and editing. **Wolfgang Sommeregger:** Funding acquisition; writing‐review and editing. **Karola Vorauer‐Uhl:** Conceptualization; supervision; writing‐review and editing.

## CONFLICT OF INTEREST

The authors declare no commercial or financial conflict of interest.

### PEER REVIEW

The peer review history for this article is available at https://publons.com/publon/10.1002/btpr.3124.

## Supporting information

**Appendix S1**: Supporting information

## Data Availability

The authors confirm that the data supporting the findings of this study are available within the article and its supplementary materials.
