# Supplementary figures and images for: Quantification of glycated IgG in CHO supernatants: A practical approach
Source: Biotechnol Prog. 2021 Jan 21;37(3):e3124. doi: 10.1002/btpr.3124 (PMC8365726; doi:10.1002/btpr.3124)

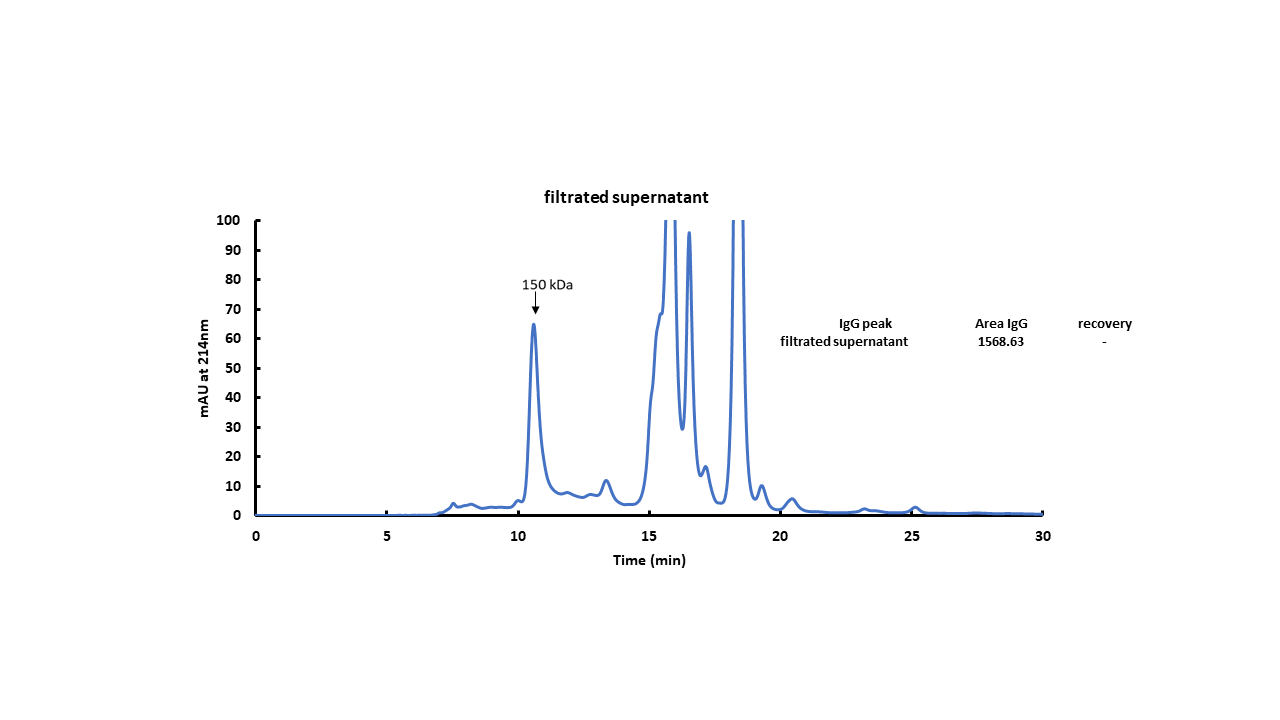

Supplement: Supplementary file 1 — Appendix S1: Supporting information [file BTPR-37-e3124-s001.zip › BTPR_3124_Supplement_S1a.tif]

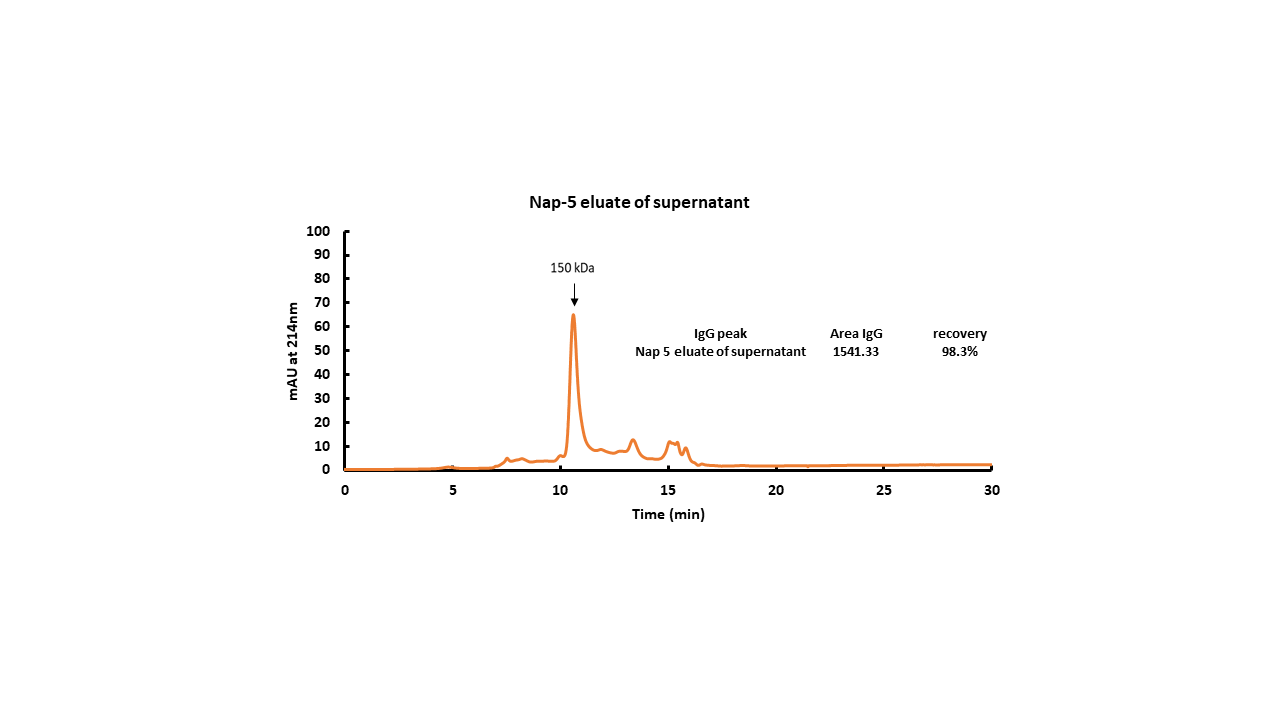

Supplement: Supplementary file 1 — Appendix S1: Supporting information [file BTPR-37-e3124-s001.zip › BTPR_3124_supplement_S1b.tif]

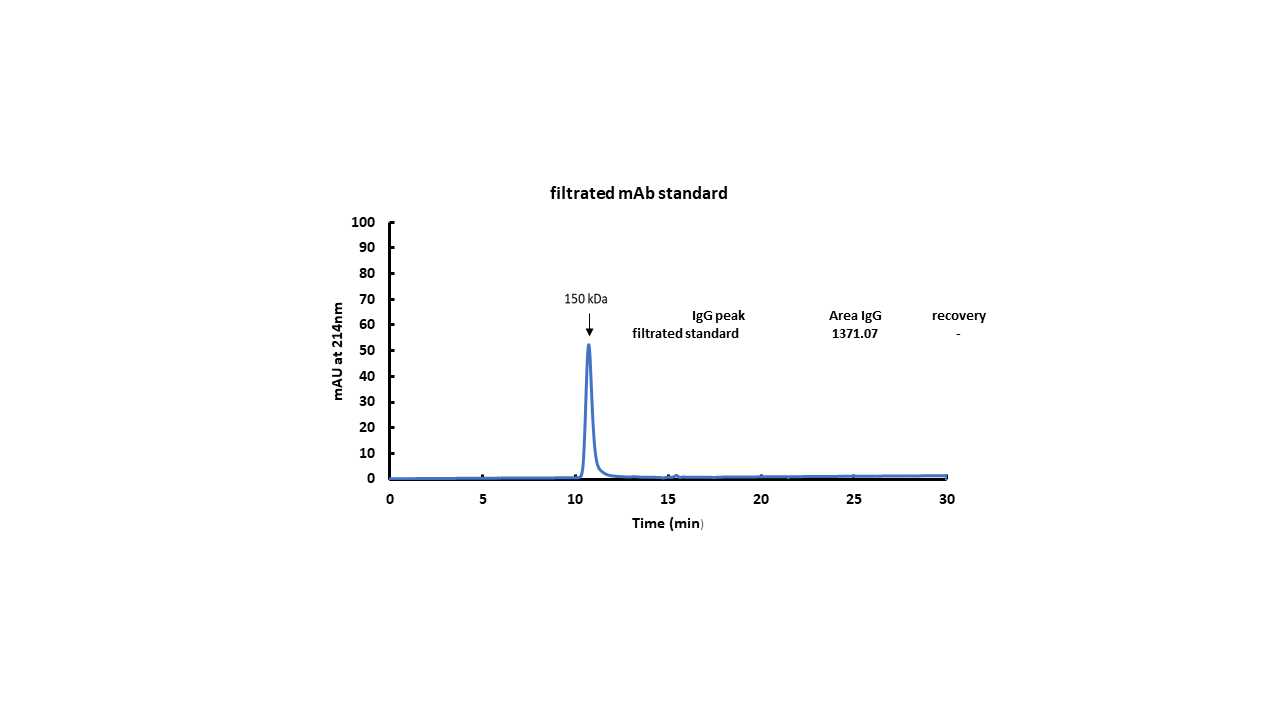

Supplement: Supplementary file 1 — Appendix S1: Supporting information [file BTPR-37-e3124-s001.zip › BTPR_3124_supplement_S2a.tif]

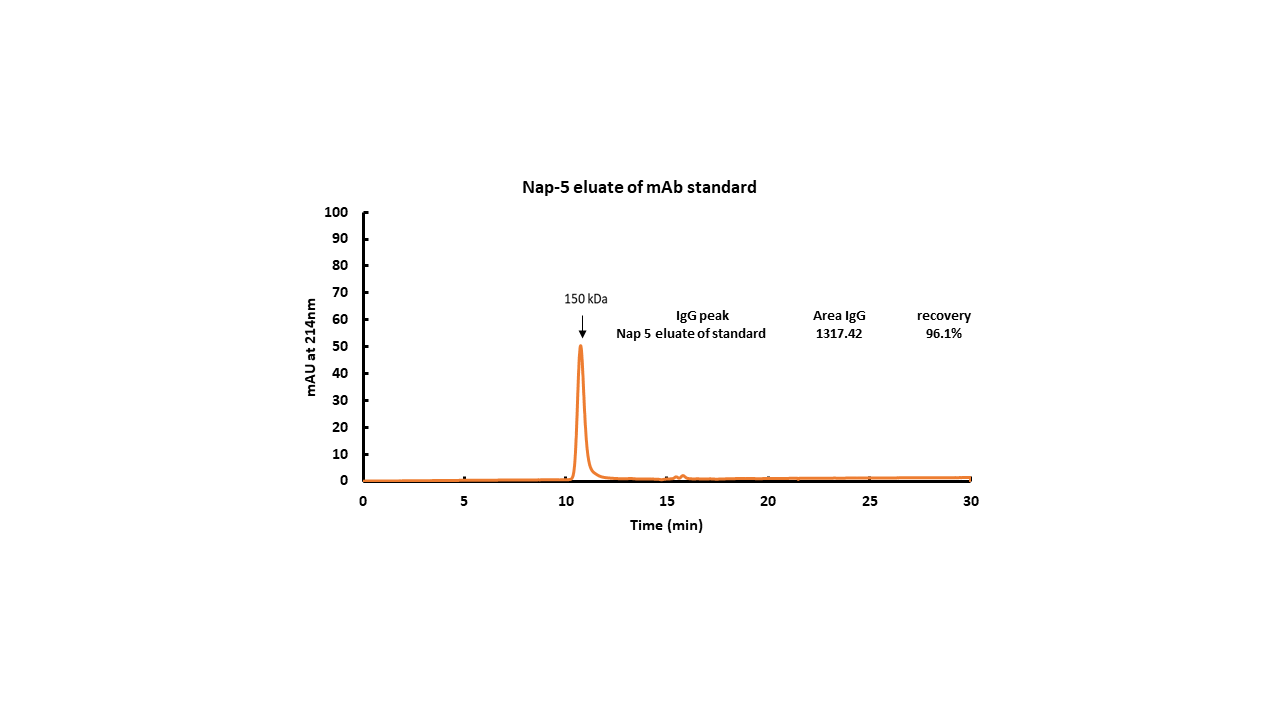

Supplement: Supplementary file 1 — Appendix S1: Supporting information [file BTPR-37-e3124-s001.zip › BTPR_3124_supplement_S2b.tif]
